# p53 Genetics and Biology in Lung Carcinomas: Insights, Implications and Clinical Applications

**DOI:** 10.3390/biomedicines12071453

**Published:** 2024-06-29

**Authors:** Dixan A. Benitez, Guadalupe Cumplido-Laso, Marcos Olivera-Gómez, Nuria Del Valle-Del Pino, Alba Díaz-Pizarro, Sonia Mulero-Navarro, Angel Román-García, Jose Maria Carvajal-Gonzalez

**Affiliations:** Departamento de Bioquímica, Biología Molecular y Genética, Facultad de Ciencias, Universidad de Extremadura, 06006 Badajoz, Spain; guadalupecl@unex.es (G.C.-L.); marcosog@unex.es (M.O.-G.); nudelvalle@unex.es (N.D.V.-D.P.); albadiazp@unex.es (A.D.-P.); smmulero@unex.es (S.M.-N.); acroman@unex.es (A.R.-G.)

**Keywords:** p53, mutated p53, lung cancer subtypes, squamous cell carcinoma, lung carcinoma, lung squamous cell carcinoma

## Abstract

The *TP53* gene is renowned as a tumor suppressor, playing a pivotal role in overseeing the cell cycle, apoptosis, and maintaining genomic stability. Dysregulation of p53 often contributes to the initiation and progression of various cancers, including lung cancer (LC) subtypes. The review explores the intricate relationship between p53 and its role in the development and progression of LC. p53, a crucial tumor suppressor protein, exists in various isoforms, and understanding their distinct functions in LC is essential for advancing our knowledge of this deadly disease. This review aims to provide a comprehensive literature overview of p53, its relevance to LC, and potential clinical applications.

## 1. Introduction

**Figure 1:**
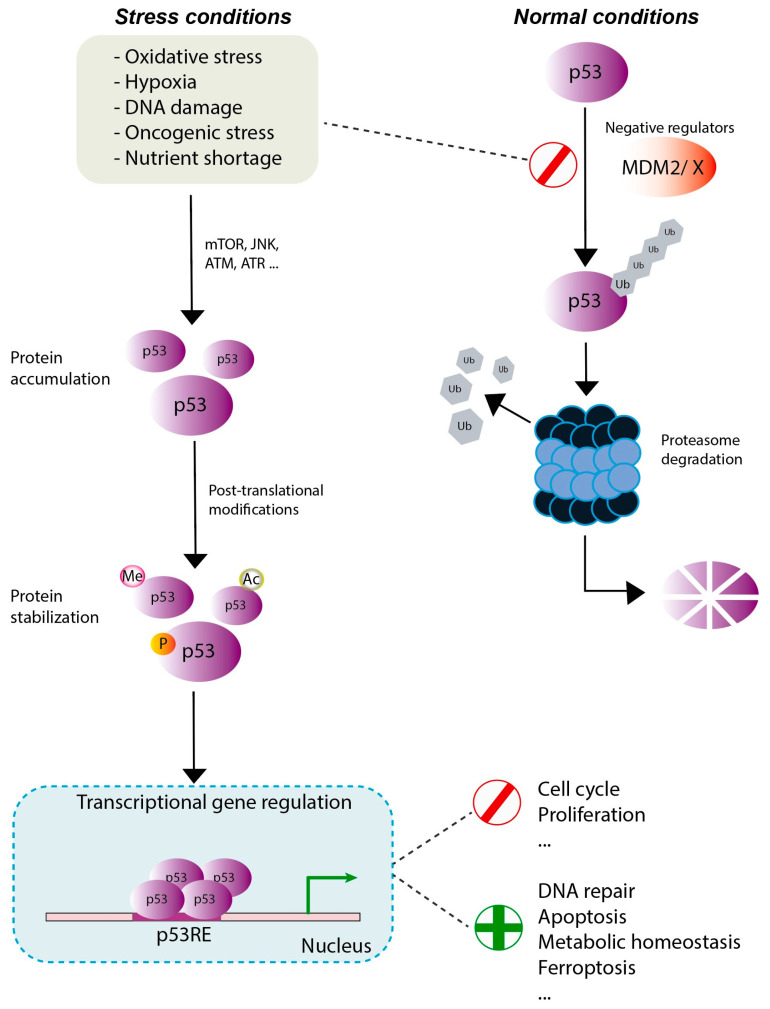
Summary overview of the p53 pathway. In normal conditions (**right** panel), the p53 protein is strongly negatively regulated by MDM2/MDMX, an E3 ligase that ubiquitinates p53, leading to its degradation in the proteasome. Under stress conditions, generated by extracellular and intracellular stress (**left** panel), p53 levels increase, and through post-translational modifications (e.g., phosphorylation, acetylation, and methylation), p53 is activated and stabilized. In the cell nucleus, p53 tetramers bind to DNA through p53 response elements (p53REs), regulating the transcription of genes that control various biological processes.

p53 protein is a critical tumor suppressor, maintaining genome stability and inhibiting tumorigenesis in the cell. The primary function of p53 is to act as a transcriptional activator for a diverse array of genes by recognizing and binding to specific DNA sequences [1]. Additionally, the p53 network plays a role in contributing to adaptive homeostasis [2,3]. Under normal conditions, p53 levels are downregulated by MDM2 and MDMX. However, in response to stimuli such as DNA damage, ribosomal stress, metabolic stress, and other conditions, p53 undergoes post-translational modifications to swiftly stabilize and subsequently activate genes with diverse cellular functions [4], as shown in Figure 1. Consequently, the p53 protein transcriptionally activates genes that play a decisive role in determining the cell’s fate—whether it survives or undergoes programmed cell death [5]. The mutation in p53 is acknowledged as one of the most common concurrent genomic alterations in non-small-cell lung cancer (NSCLC). Despite the establishment of an oncogene-centric molecular classification paradigm for this lung cancer [6], the precise role of p53 in NSCLC remains unclear, particularly in two of its primary subtypes with distinct cellular origins, namely, lung adenocarcinoma (LUAD) and lung squamous cell carcinoma (LUSC).

### Other Relevant Tumor Suppressors

p53, Rb1 (retinoblastoma 1), and PTEN (phosphatase and TENsin homolog) are three key tumor suppressor genes that play critical roles in maintaining genomic stability and preventing the development of cancer. While they share some common functions, each tumor suppressor also possesses unique characteristics that contribute to their specific roles in cancer biology. As mentioned before, p53 is often referred to as the “guardian of the genome” due to its central role in regulating cell cycle progression, DNA repair, apoptosis, and senescence in response to various cellular stresses, including DNA damage and oncogenic signaling. It acts as a transcription factor, regulating the expression of genes involved in these processes [7,8,9,10,11].

Rb1, on the other hand, functions primarily in regulating the cell cycle by inhibiting the activity of the E2F family of transcription factors. By binding to E2F, Rb1 prevents the expression of genes required for S-phase entry, thereby halting cell cycle progression and promoting cell cycle arrest or apoptosis. Dysfunction of Rb1 is associated with a wide range of cancers, particularly retinoblastoma, osteosarcoma, and small-cell lung cancer [12]. PTEN functions primarily as a lipid phosphatase that antagonizes the PI3K/AKT/mTOR signaling pathway, thereby regulating cell growth, proliferation, and survival. By dephosphorylating phosphatidylinositol (3,4,5)-trisphosphate (PIP3), PTEN suppresses downstream signaling events that promote cell survival and proliferation. Loss of PTEN function is frequently observed in various cancers, including glioblastoma, prostate cancer, and endometrial cancer [13]. While all three tumor suppressors play crucial roles in preventing cancer development, they exhibit both overlapping and distinct functions. For example, p53 and Rb1 both regulate cell cycle progression, albeit through different mechanisms, whereas PTEN primarily regulates cell growth and survival signaling pathways. Additionally, all three tumor suppressors can induce apoptosis, although p53 is perhaps best known for its role in this process. Dysfunction of any of these tumor suppressors can lead to uncontrolled cell proliferation and tumorigenesis, underscoring their importance in maintaining cellular homeostasis and preventing cancer [11,14,15,16].

## 2. p53: Gene and Protein

The *TP53* tumor suppressor gene (NG_017013) is situated in chromosome 17 at the short arm (17p13.1) and comprises 13 exons, with the first being noncoding [17]. Highly conserved in multicellular organisms [18], the *TP53* gene exhibits numerous genetic polymorphisms, describing over 100 different haplotypes of *TP53*. Some of these haplotypes are associated with an elevated risk of cancer [19,20,21]. Previous studies have identified *TP53* as the most frequently mutated gene in human cancers [22,23,24,25,26,27,28,29]. Despite this recognition, translating *TP53* mutation status into effective cancer treatment and predicting clinical outcomes remains challenging in the clinical setting. This highlights that our comprehension of the p53 pathway is not yet exhaustive. The identification of various splice variants encoded by the *TP53* gene may provide an explanation for this discrepancy [17].

The *TP53* gene stands out as one of the most extensively studied genes in recent decades [30]. For many years, it was commonly believed that human *TP53* exclusively expressed a single protein, p53. However, in 2005, Burdon et al. revealed that the *TP53* gene expresses a minimum of 12 isoforms of p53 in humans [31]. *TP53* is expressed not only as the full-length p53 (FLp53, canonical p53, wild-type p53, or p53α) but also as 11 smaller isoforms: p53β, p53γ, ∆40p53α, ∆40p53β, ∆40p53γ, ∆133p53α, ∆133p53β, ∆133p53γ, ∆160p53α, ∆160p53β, and ∆160p53γ [31,32,33]. These isoforms are produced via processes such as alternative splicing, alternative initiation of translation, alternative promoter usage, or post-translational degradation of p53 through the 20S proteasome [31,32,34,35,36].

At the protein level, full-length p53 is characterized by distinct functional domains. These include two transactivation domains (TADs) at the N-terminus, a proline-rich domain (PRD), a central DNA-binding domain (DBD), an oligomerization domain (OD), and a regulatory C-terminal domain (CTD) at the carboxy-terminal region [37]. The N-terminal segment of the protein encompasses the transactivation domain, housing regions that engage in protein–protein interactions with regulatory proteins, such as MDM2. This interaction initiates ubiquitination, ultimately leading to the degradation of the protein in the proteasome [38,39,40].

At the functional level, the p53 protein acts as a transcription factor contributing to tumor suppression by activating the expression of a multitude of target genes [37]. In the context of cancer, p53 plays a crucial role in restraining cell proliferation in response to a variety of stimuli, which include factors like DNA damage, nutrient scarcity, hypoxia, and hyperproliferative signals, so this function of p53 acts as a barrier against the formation of tumors [41], earning it the well-deserved title of the guardian of the genome. Under normal physiological conditions, MDM2, an E3 ligase, meticulously controls the levels of p53, initiating its degradation through the ubiquitin proteasome-dependent pathway. Furthermore, p53 can induce the expression of MDM2, recognizing and interacting with its promoter region, establishing a negative regulation designed to promote the degradation of p53 and maintain low cellular p53 levels [42]. When the cell faces various stress situations, p53 is subjected to several post-translational modifications, notably phosphorylation, which serves to stabilize p53 as it interferes with the interaction with MDM2. In addition, sequence-specific DNA binding and transactivation may take place. Through these processes, p53 either activates or suppresses target genes involved in regulating the cell cycle, DNA repair, and the induction of senescence and apoptosis [43].

In addition to its dominant-negative activity, which interferes with the functions of wild-type p53, numerous studies have demonstrated that mutations in p53 can afford oncogenic properties through gain-of-function (GOF) mechanisms. In these scenarios, the mutant p53 protein engages with new transcription factors or cofactors, exerting control over gene transcription and expression to facilitate the development of cancer [23,28,44,45,46,47,48,49,50,51].

The *TP53* gene has been implicated in an expanding array of biological processes, encompassing cell cycle arrest, apoptosis, DNA repair, autophagy, metabolism, senescence, and aging [1,52,53,54,55,56]. The zinc ion plays a pivotal role as a cofactor for wild-type p53, crucial for its effective binding to DNA. Any mutations affecting the zinc-coordinating residues disrupt the ability of p53 to bind to zinc ions, rendering it incapable of interacting with DNA [57].

Some studies have demonstrated dysregulation in the mRNA expression of p53 isoforms in various cancers [31,58,59,60,61,62,63,64], where the p53 isoforms have been associated with prognosis [62,65] and chemotherapy response [58,59].

Efforts to destabilize mutant p53 and thwart its GOF activities are actively explored as a hopeful therapeutic strategy for cancers with p53 mutations [23,48,66,67,68]. However, the precise mechanisms governing the accumulation of mutant p53 in cancer remain incompletely understood, posing a challenge to the development of effective strategies for treating cancers carrying mutant p53 [69]. Presently, there are no clinically available drugs that target mutant p53 for cancer treatment [23,67,68]. Therefore, gaining a more thorough understanding of the mechanisms responsible for the accumulation of p53 mutants and their GOF activities in cancer is crucial [69].

The most common polymorphisms in the *TP53* gene include the following. (1) rs1042522 (p.Arg72Pro or R72P): This polymorphism results in an arginine (Arg)-to-proline (Pro) substitution at codon 72 of exon 4. The Arg72 variant is associated with increased apoptosis and has been linked to a higher risk of certain cancers, such as lung cancer and colorectal cancer. The Pro72 variant is less efficient in inducing apoptosis but may be associated with other cancers like breast cancer. (2) rs1625895 (intron 3): This polymorphism is found in intron 3 of the *TP53* gene and can affect splicing or gene regulation. Certain intronic polymorphisms are linked to different cancer risks depending on the population studied. (3) rs17878362 (16-bp Ins/Del): This is a 16-base pair insertion/deletion polymorphism in intron 3. This variation can influence *TP53* mRNA splicing and is associated with altered cancer risk [70,71,72].

Specific haplotypes formed by these polymorphisms can either increase or decrease susceptibility to various cancers depending on the population and the type of cancer. Understanding these genetic variations and their functional consequences helps in predicting cancer risk and developing targeted therapies. The Pro72 variant combined with specific intron 3 polymorphisms (such as rs1625895) forms a haplotype that has been linked to a higher risk of developing cancer, particularly in Asian populations [73,74]. The Arg72 variant, when associated with certain other polymorphisms in the *TP53* gene, forms a haplotype that might be protective in some cancers but risk-enhancing in others. For example, the Arg72 variant has been associated with increased risk of colorectal cancer but may be protective against other types like bladder cancer. Haplotypes involving the 16-bp insertion/deletion polymorphism (rs17878362) in combination with other *TP53* polymorphisms can influence cancer risk. The specific combination can either increase susceptibility or confer protection depending on the cancer type [19,73,75,76,77,78,79,80].

## 3. LC and p53

*TP53* is the most frequently mutated gene in human cancers because it regulates the cell cycle, promotes DNA repair, and initiates apoptosis in response to DNA damage. This makes it a critical barrier to cancer development, as the inactivation of p53 removes a major obstacle to uncontrolled cell division. Tumors often evolve to bypass the growth-inhibitory effects of p53. In addition, the *TP53* gene is particularly susceptible to damage from various environmental mutagens and carcinogens. This high exposure increases the likelihood of mutations, and mutations in *TP53* can simultaneously disrupt multiple tumor suppressive pathways, including cell cycle arrest, apoptosis, DNA repair, and senescence. This broad impact makes *TP53* mutations particularly advantageous for cancer cells [81,82,83].

The main types of *TP53* mutations and their effects on cancer development are listed as follows.

Missense Mutations: A single nucleotide change results in the substitution of one amino acid for another in the p53 protein. These mutations often occur in the DBD, leading to a loss of DNA-binding ability and the subsequent loss of transcriptional regulation of target genes. Many missense mutations produce a dominant-negative effect, where the mutant p53 interferes with the function of any remaining wild-type p53, or a GOF effect, where the mutant protein acquires new oncogenic properties. Nonsense Mutations: A single nucleotide change introduces a premature stop codon, resulting in a truncated, non-functional p53 protein. These mutations usually lead to complete loss of p53 function (LOF), as the truncated protein is typically unstable and degraded rapidly, preventing it from exerting any tumor suppressive effects. Frameshift Mutations: Insertions or deletions of nucleotides alter the reading frame of the gene, leading to the production of an aberrant protein. Frameshift mutations generally produce non-functional p53 proteins that are either truncated or have lost critical domains necessary for tumor suppression. These proteins are often degraded or fail to perform their regulatory functions. Splice Site Mutations: Mutations at the junctions of exons and introns affect the splicing of *TP53* mRNA. These mutations can lead to the production of abnormal p53 isoforms that either lack essential functional domains or have altered regulatory properties, potentially contributing to cancer progression. Intragenic Deletions: Deletions within the *TP53* gene remove entire exons or parts of exons. These deletions often result in truncated p53 proteins that are non-functional and unstable, leading to a complete loss of p53 tumor suppressive activity [81,82,83,84,85,86,87,88].

LC stands as the foremost cause of cancer-related deaths globally [89,90]. As mentioned above, lung cancer is divided into two primary types: small-cell lung cancer (SCLC) and NSCLC. Each year, approximately 2 million new cases of lung cancer are diagnosed, leading to 1.8 million global deaths attributed to the disease [91]. Histopathologically, NSCLC is the predominant type, accounting for 80–85% of all lung cancer cases [92,93,94]. LUSC constitutes around 30% of NSCLC cases, making it the second most prevalent histological type of lung cancer [95].

Dysregulation of p53 often contributes to the initiation and progression of various cancers, including LUSC. In the initiation phase, loss of cell cycle control due to mutations or loss of p53 disrupt its function in arresting the cell cycle and facilitating DNA repair, potentially allowing cells with DNA damage to propagate, initiating tumorigenesis [96,97]. The compromised p53 can reduce apoptosis, permitting survival of transformed cells that might otherwise be eliminated. In this way, p53 inactivation may result in reduced apoptotic potential, allowing the survival of cells with aberrant growth characteristics [98]. In the progression phase, the p53 dysregulation fosters genomic instability, enabling accumulation of additional mutations that may facilitate cancer progression [99]. Altered p53 can influence angiogenesis, fostering a tumor-permissive microenvironment that supports progression. Some studies suggest that p53 mutations may be linked to enhanced invasive and metastatic potential in, e.g., head and neck squamous cell carcinoma, although the precise mechanisms remain to be fully elucidated [100]. Also, mutant p53 can induce epithelial–mesenchymal transition and metastasis, enhancing the migratory and invasive properties of tumor cells [101]. p53 mutations might interact with other oncogenic pathways (like PI3K/AKT and MAPK), further supporting tumor progression and resistance to therapy [102], and may also influence the tumor microenvironment through immune regulatory pathways, potentially enabling tumors to evade immune surveillance [103]. Recently, it has been reported that the regulation of p53 by MDM2 plays an active role in regulating both the self-renewal and differentiation processes of basal stem cells in both mouse and human airway epithelium [104]. On the other hand, a recent publication analyzing Trp53 null and wild-type mice demonstrated that p53 plays a role in directing alveolar regeneration following injury. It achieves this by regulating alveolar type 2 (AT2) cell self-renewal and promoting the transitional differentiation of cells into alveolar type 1 (AT1) cells. The reported results could provide insights into the mechanisms of p53-mediated suppression of LUAD. In this context, p53 appears to play a pivotal role in regulating alveolar differentiation, suggesting that the suppression of tumors might be tied to the essential role of p53 in coordinating tissue repair after injury [105].

The mutation in p53 is acknowledged as one of the most common concurrent genomic alterations in NSCLC. This observation persists despite the establishment of an oncogene-centered molecular classification paradigm for this type of lung cancer [6]. The prevalence of *TP53* mutation exceeds 90% in LUSC, and concurrently, the tumor suppressor cyclin-dependent kinase inhibitor 2A (CDKN2A), responsible for cell cycle regulation, is inactivated in approximately 70% of LUSC cases [106]. Nevertheless, the distinct function of p53 in NSCLC, particularly in its major subtypes with distinct cellular origins—LUAD and LUSC—are still to be clarified. Several studies suggest that mutant p53 correlates with unfavorable clinical outcomes [107,108,109], while the activation of p53 signaling or restoration of wild-type p53 has been demonstrated to suppress tumorigenesis [110,111,112]. Nevertheless, elevated expression of the oncoprotein p53 has been proposed as a promising prognostic indicator in a subgroup of patients with NSCLC [113]. In addition, recent research indicates that the existence of mutant p53 is correlated with a shorter survival outcome in patients diagnosed with LUAD and, conversely, in those patients diagnosed with LUSC, significantly longer overall survival [114]. Enhancing our comprehension related to the function of p53 in the progression of these lung cancer subtypes holds the promise of developing a more targeted and rational therapeutic strategy, potentially leading to an extension in the survival of the patient. Despite LUAD and LUSC presenting a distinct biological imprint, their treatment approaches often overlap [94]. In a recent study, it was observed that p53 expression in LUAD and LUSC is linked to distinct activation or suppression pathway profiles. Despite the historical perception of p53 as challenging to directly target, the pathways influenced by p53 expression might offer viable targets for cancer therapy. Additionally, understanding the unique pathways in LUAD or LUSC may guide the development of more precise and subtype-specific cancer therapies in the coming years [115].

The correlation between lung cancer and tobacco use is intricate, with variations observed among histologic subtypes. LUAD primarily manifests in individuals without significant tobacco exposure, while LUSC is predominantly found in current or former smokers [116]. Adding complexity to this association, changes in the composition of cigarettes are believed to contribute to differences not only in the risks associated with smoking and lung cancer but also in the histologic subtypes linked to tobacco exposure [117,118].

The hallmark of cancer often involves significant changes in the epigenetic landscape. Specifically, in LUSC, frequent mutations are observed in genes that encode epigenetic regulators [119,120,121]. Significantly, *TP53* mutations were linked to the emergence of new distant metastases. Notably, these mutations were more prevalent among patients with a history of smoking, implying an increased risk of distant metastasis in individuals with such a smoking history [122].

Mutational hotspots are primarily concentrated within the sequence-specific DNA-binding domain, with approximately 75% of mutations being missense alterations, resulting in the loss of its function as a transcription factor [123]. The mutational patterns are influenced by smoking [123,124], and a correlation exists between p53 mutational hotspots and sites of adduct formation by polycyclic aromatic hydrocarbon [125,126]. The build-up of p53 non-functional mutants contributes to increased concentrations of mutated p53 in tumor cells [127].

**Figure 2:**
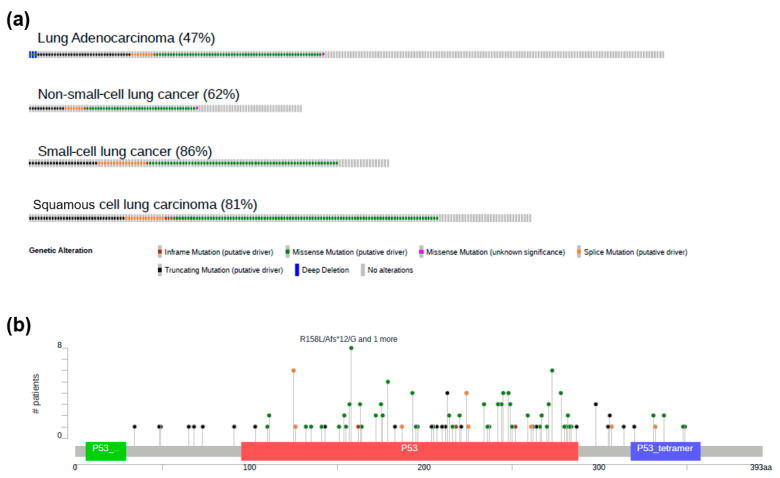
Genetic alterations of p53 in the different types of lung cancer from cBioPortal datasets. (**a**) Distribution of p53 mutation type in LUAD (N = 221), NSCLC (N = 240), SCLC (N = 110), and LUSC (N = 175). (**b**) Point mutations within the p53 gene in LUSC patients. Green, red, and violet boxes correspond to transactivation domain, DNA-binding domain, and oligomerization domain, respectively.

Utilizing cBioPortal for Cancer Genomics [128,129,130], an open access, open source resource facilitating interactive exploration of multidimensional cancer genomics datasets, we conducted an analysis of the genetic alterations of p53 in various types of lung cancer. As shown in Figure 2a, all lung cancer subtypes show alterations in the p53 gene to different degrees: LUAD 47%, NSCLC 62%, LUSC 81%, and SCLC 86%. Among all the alterations described, putative-driven missense mutations (green tags) occur most predominantly, being particularly important in LUSC, where they account for most of all genetic alterations.

When analyzing the point mutations within the p53 gene in LUSC, mutations are concentrated in the DNA-binding domain (red) with R158L/Asf*12/G being the most abundant among all patients (Figure 2b). Note that missense mutations are also the most common in this domain. Taking together the data in Figure 2a,b, it can be observed that the majority of these missense mutations are located along the DNA-binding domain segment of the p53 gene.

### LOF and GOF of p53 in LC

In lung cancer, alterations in the p53 pathway are prevalent, with both LOF of wild-type p53 and GOF promoted by p53 mutants contributing to tumorigenesis and disease progression. Wild-type p53 functions as a tumor suppressor by inducing cell cycle arrest or apoptosis in response to cellular stress, thereby preventing the propagation of damaged cells [131,132]. LOF of p53 is critical for the proliferation, survival, and metastasis of a broad range of cancer cells, including lung cancer [133]. LOF enhances the metastatic potential of LUSC cells through the dysregulation of epithelial–mesenchymal transition markers [134]. Inactivating mutations of the TP53 gene results in the LOF of wild-type p53 and/or dominant-negative p53 mutants. In this respect, studies using mice as the model suggest that certain p53 mutations confer oncogenic GOF activities that promote tumorigenesis and metastasis [135,136]

**Figure 3:**
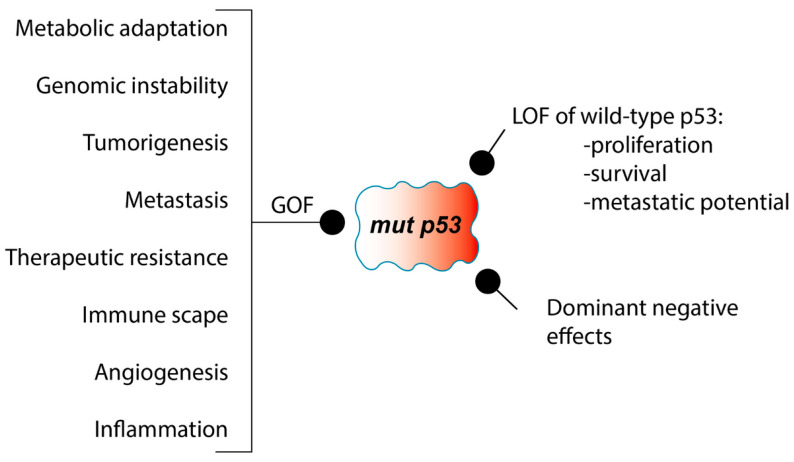
Mutant p53 in cancer. Mutations of p53 can lead to various oncogenic effects, referred to as GOF, among which are metabolic adaptations, genomic instability, tumorigenesis, metastasis, and increased resistance to anticancer treatments. Negative dominant effects on the wild-type protein as well as LOF of the wild-type p53 protein include promoting proliferation, survival, and conferring metastatic potential to cells.

In contrast to wild-type p53, mutant forms of p53 frequently exhibit GOF properties, conferring oncogenic functions that drive tumorigenesis and therapeutic resistance [137], as shown in Figure 3. In lung cancer, specific p53 mutations are associated with distinct GOF activities, including increased cell proliferation, invasion, and chemoresistance. The majority of gene mutations manifest as missense mutations, primarily found within the DNA-binding functional domain of the p53 gene. Notably, the mutation frequency was highest for six specific amino acid residues in p53: R175, G245, R248, R249, R273, and R282 [138,139]. These mutations allow the p53 protein to potentially engage with non-canonical protein partners, thus facilitating oncogenesis. Interesting, recent data suggest that chloroquine treatment resulted in cytoplasmic accumulation and reduced transcriptional activity of GOF p53 R273H and YAP (Yes-associated protein), leading to growth arrest of NSCLC cells [140].

## 4. p53: Clinical Applications and Therapies

Addressing the p53 axis has posed challenges because p53, being a transcription factor, involves intricate protein–protein interactions. Unlike more easily druggable targets with accessible receptor–ligand interactions or enzymatic active sites, p53 lacks such characteristics, adding complexity to its therapeutic targeting [141]. Diverse approaches have been explored to target p53, ranging from adenovirus-based gene therapy to the recent development of small molecules designed to activate endogenous p53 in tumors that retain the wild-type p53 gene [127]. A potential strategy involves the development of small molecules to boost p53 activity by neutralizing MDM2, such as nutlins, which bind to MDM2 and dissociate it from p53. Additionally, there are ongoing efforts to develop small molecules that target mutant p53. However, this is a huge challenge due to the diverse array of expressed p53 mutant proteins [141]. Therefore, p53 is a challenging protein to target for the development of inhibitors. Nonetheless, progress in unraveling the structure of p53 and its interactions with partners has opened the door to the exploration and development of a multitude of molecules that hold promise to reinstate the tumor-suppressing functions of p53 [43].

As indicated by various studies, the observed widespread inactivation of p53 is a prevalent characteristic in NSCLC [142,143,144,145]. This suggests that the restoration of p53’s anticancer function with a p53 activator may represent a hopeful therapeutic strategy for treatment in patients with NSCLC [146,147,148,149]. Some p53 small-molecule activators have been developed and investigated with the aim of restoring the functionality of the p53 protein. Several of these hopeful compounds are currently being subjected to clinical trial evaluation. In this regard, encouraging therapeutic outcomes have been noted in preclinical and clinical studies involving the treatment of solid and hematological tumors with p53 activators specifically designed to disrupt the MDM2/X–p53 interaction [150,151,152].

For example, Kevetrin, (thioureidobutyronitrile or 3-cyanopropyl carbamimidothioate hydrochloride, C5H10ClN3S) is a small-molecule compound displaying activity both dependent on and independent of p53. It has demonstrated favorable tolerability and therapeutic potential across a spectrum of solid tumors, encompassing lung, breast, colon, and ovarian cancers [153,154,155,156].

Furthermore, the development of a synthetic small-molecule p53 activator named NA-17, whose action would be mediated by reorganization of the Bak-Bcl-Xl complex and activation of transcriptional regulation, has shown hopeful results in preclinical models of NSCLC [157]. Nevertheless, in normal cells and in oncogene-driven tumors, this compound has demonstrated relatively high toxicity and limited therapeutic efficacy, respectively. There is potential for obtaining p53 activators with both high efficiency and low toxicity through the optimization of the lead molecule [158]. In their study, they conducted high-performance screening of optimized compounds from NA-17, with the aim of finding new activators of p53. MX-C2 and MX-C3 molecules were discovered, and as noteworthy candidates, both compounds demonstrating substantial therapeutic efficacy in oncogene-driven tumor models. Like NA-17, these compounds were able to induce the activation of p53 by phosphorylating serine-392 without inducing DNA damage. Interestingly, in NSCLC cells and in control cell lines, both molecules exhibited extensive antitumor activity and reduced toxicity, respectively [158].

LUAD stands as the second most common category, comprising 20% to 30% of deaths attributed to lung carcinoma [116,159]. However, unlike LUAD, for which targeted therapies including the anaplastic lymphoma kinase (ALK) and epidermal growth factor receptor (EGFR) inhibitors have proven significantly effective [160], there is currently no approved vanguard targeted therapy for the management of individuals diagnosed with LUSC [161,162,163].

In recent times, interventions utilizing immune checkpoint blockades through antibodies that hinder inhibitory immune control proteins, like programmed cell death protein 1 (PD-1) or its ligand (PD-L1), have surfaced as pivotal elements in the established treatment protocol for LUSC patients. However, despite these advancements, the rate of response remains modest. Hence, the identification of potent therapeutics stands as a crucial and pressing unmet requirement for individuals diagnosed with LUSC [164,165]. Targeting of the Notch ligand DLL3 has shown promise in the development of innovative approaches for LUSC, yielding encouraging initial outcomes [106]. For more details on the transcriptional regulation of airway epithelial cell differentiation and the role of Notch, see [166]. Notwithstanding meticulous genomic examination, discerning the oncogenic catalysts in LUSC persists as a formidable challenge [116,120].

Recently, Niu et al. conducted a review on advancements in epigenetic treatments, immune checkpoint inhibitors (ICIs), and diverse combination approaches involving ICIs and additional targeted interventions for LUSC. The review also delved into the potential opportunities and obstacles associated with exploring and implementing innovative therapeutic approaches for LUSC [167]. An initial-phase clinical trial is investigating vaccination of tumor using the *TP53*-DC vaccine in conjunction with nivolumab and ipilimumab in SCLC. Additional approaches aiming to enhance the immunogenicity of tumor through targeted therapies, including histone deacetylase (HDAC), DNA methyltransferases (DNMTs), or poly-ADP ribose polymerase (PARP) inhibitors, integrated with ICIs, are also undergoing evaluation in initial-phase clinical investigations [106].

*TP53* mutations significantly affect the response to standard chemotherapy in lung cancer patients. For example, *TP53* mutations confer resistance to platinum-based chemotherapy, taxanes, and etoposide [168,169,170,171,172].

The *TP53*-associated signature is a specific and independent prognostic biomarker for LUSC patients and could provide potential prognostic biomarker or therapeutic targets for the development of novel immunotherapies and chemotherapies [173].

Several novel targeted therapies are being developed to specifically target *TP53*-mutated LCs [174]. These therapies focus on reactivating or bypassing the dysfunctional p53 pathway, exploiting synthetic lethality, and combining existing treatments to enhance efficacy, for example, APR-246, PLK4-inhibitors, and mitophagy inhibitors [171,173,175,176]. On the other hand, there are some biomarkers and clinical features that can predict the likelihood of *TP53* mutation in LC patients, for instance, KRAS and *TP53* co-mutation, *TP53* mutations in normal airway epithelium, tumor mutation burden, smoking history, imaging, and radiomics [177,178,179,180,181,182,183,184].

**Figure 4:**
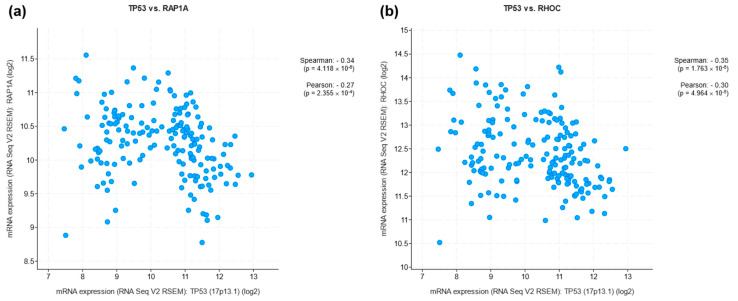
Relation of *TP53* gene expression in LUSC patients from cBioPortal datasets with RAP1A gene expression (**a**) and RHOC gene expression (**b**).

*TP53* gene expression in LUSC patients was analyzed with the expression of several genes from cBioPortal datasets. This analysis revealed that the expression of RAP1A and RHOC genes is most closely related to p53 gene expression (Figure 4a and Figure 4b, respectively). Ras-associated protein 1A (Rap1A) and Ras homolog family member C (RhoC) are small GTP-binding proteins categorized within the Ras subfamily. These proteins exhibit a dynamic transition between an inactive GDP-bound state and an active GTP-bound state [185,186]. While Ras and Rap1A share nearly identical effectors in the vicinity of the cell surface, there is a distinction in their activation locations. Rap1A undergoes activation in the perinuclear region, in contrast to most Ras proteins, which undergo activation at the plasma membrane [187]. p53 regulates RhoC transcription and activation [188], and as for Rap1A, it has been proposed that there is a putative binding site of Rap1A to p53 [189]. Both proteins could be promising targets in patients with LUSC.

Mutations in the *TP53* gene are common in NSCLC and have significant implications for clinical outcomes. The correlation between p53 mutations and clinical outcomes can vary between the two principal subtypes (LUAD and LUSC) due to differences in their biology and mutation profiles [89,120].

In LUAD, *TP53* mutations are generally associated with a worse prognosis. Patients with *TP53* mutations tend to have lower overall survival rates compared to those without these mutations. Studies have shown that *TP53* mutations in LUAD are linked to higher tumor grade, increased metastasis, and resistance to certain therapies, contributing to poorer outcomes. *TP53*-mutant LUAD may exhibit resistance to traditional chemotherapies and targeted therapies, making treatment more challenging. The presence of *TP53* mutations can influence the effectiveness of emerging treatments such as immunotherapy. Some studies suggest that *TP53* mutations might be associated with better responses to ICIs, although this is an area of active research, and findings are not yet conclusive [190,191].

In LUSC, TP53 mutations are also associated with poor clinical outcomes. Similar to LUAD, patients with *TP53*-mutant LUSC often have a worse prognosis, with lower overall survival rates and higher rates of disease recurrence. The high prevalence of *TP53* mutations in LUSC contributes to its aggressive nature and poor response to standard treatments. *TP53* mutations in LUSC are linked to resistance to conventional chemotherapy and radiation therapy, contributing to poorer clinical outcomes. As with LUAD, there is ongoing research into the impact of *TP53* mutations on the response to immunotherapy in LUSC. Some studies suggest that these mutations might be predictive of a favorable response to ICIs [190,191].

The frequency of *TP53* mutations varies significantly across different subtypes of LC. The two major subtypes of NSCLC, LUAD and LUSC, each show distinct patterns of *TP53* mutations. Additionally, SCLC, a less common but more aggressive type of LC, also displays a unique pattern of *TP53* mutations. *TP53* mutations in LUAD often involve missense mutations, particularly in the DBD, which lead to the production of a dysfunctional p53 protein. Other types of mutations, such as nonsense and frameshift mutations, are also present but less common. Similar to LUAD, missense mutations are the most prevalent type of *TP53* mutation in LUSC. These mutations typically affect the DBD and result in loss of p53 function. Nonsense and frameshift mutations also affect the DBD. SCLC is characterized by a high frequency of both missense and truncating mutations (nonsense and frameshift), leading to complete loss of p53 function. The mutation pattern in SCLC often involves extensive genomic instability [89,94,120,190,192,193,194].

cBioPortal analysis provides a comprehensive view of *TP53* alterations in LC subtypes, revealing their prevalence, types, and functional impacts. These insights help us understand the crucial role of *TP53* in lung cancer biology, highlighting the importance of p53 in maintaining genomic stability and suppressing tumor development. Furthermore, the patterns of *TP53* mutations observed in cBioPortal data inform our understanding of prognosis and therapeutic responses, offering potential avenues for targeted treatments and personalized medicine approaches in lung cancer [128,130,190,192,193,194,195,196].

Targeting *TP53* mutations in LC treatment presents several challenges due to the complex nature of the *TP53* gene and its critical role in regulating cell growth and apoptosis. Researchers are actively working to address these challenges through various innovative approaches. Here are the main challenges and strategies being employed. High mutation diversity: *TP53* mutations are highly diverse, with hundreds of different mutations identified in various cancers. This diversity makes it difficult to develop a one-size-fits-all treatment [9]. LOF: Many *TP53* mutations result in a complete loss of tumor suppressor function, making it challenging to restore normal p53 activity. Resistance mechanism: *TP53* mutations can confer resistance to conventional therapies such as chemotherapy and radiation, complicating treatment regimens. Detection and monitoring: Accurately detecting and monitoring *TP53* mutations in tumors can be difficult due to the heterogeneous nature of LC and the mutation profile in each case [174,197,198,199,200,201,202].

There are emerging strategies for early detection and prevention of *TP53*-driven lung cancers, for example, advanced molecular profiling and liquid biopsies. Advanced molecular profiling, including NGS, is crucial for early detection of *TP53* mutations in LC. By analyzing tumor DNA from tissue samples, clinicians can identify specific mutations that drive cancer progression. NGS allows for comprehensive genomic profiling, facilitating the identification of *TP53* mutations even at early stages of cancer development [203,204]. Liquid biopsies offer a non-invasive alternative for detecting *TP53* mutations by analyzing ctDNA in blood samples. This method enables continuous monitoring of mutation status, providing early indications of cancer development or progression without the need for repeated tissue biopsies [10]. Chemoprevention and lifestyle interventions aim to either prevent the occurrence of mutations or mitigate their effects once they occur. For instance, antioxidants and other compounds that reduce oxidative stress may help in reducing mutation rates. Preventive strategies also include lifestyle modifications, particularly smoking cessation. Smoking is a major risk factor for *TP53* mutations in lung cancer, and quitting smoking significantly reduces the risk [205,206,207,208,209,210]. Early screening programs are another current strategy. Screening programs targeting high-risk populations, such as long-term smokers and individuals with a family history of lung cancer, are being implemented to detect early signs of *TP53* mutations. Also, genetic screening for individuals with a hereditary predisposition to *TP53* mutations (e.g., those with Li–Fraumeni syndrome) can help in early detection and preventive measures [211,212,213,214,215,216,217,218,219]. Last but not least are targeted therapies and immunoprevention. These therapies aim to either restore normal p53 function or exploit vulnerabilities in *TP53*-mutated cells. For example, small molecules that can reactivate mutant p53 proteins or induce synthetic lethality are under investigation [220,221,222,223,224,225]. Immunopreventive strategies involve using vaccines or immune-modulating agents to prevent the development of *TP53*-driven cancers. By enhancing the immune system’s ability to recognize and eliminate precancerous cells with *TP53* mutations, these strategies aim to prevent cancer before it fully develops [226,227,228,229,230,231,232].

Even with recent advances, the molecular basis of lung carcinogenesis needs to be further studied to better understand the molecular pathways involved in lung tumorigenesis. Since p53-related genetic alterations are a common denominator in all cancers, including lung cancer, further study of the molecular alterations of p53 is necessary to understand how this protein exerts its antitumor activity. These studies are of great value for the development of new cancer treatments using highly effective methods directed exclusively against cancer cells. Understanding the role of p53 in LUSC, both in terms of initiation and progression, has potential therapeutic implications. For example, strategies aimed at restoring p53 function or targeting the consequences of p53 mutations could provide avenues for novel LUSC therapies. Furthermore, considering p53 status in the context of personalized medicine approaches could facilitate more effective and individualized therapeutic strategies for LUSC patients.

## 5. Conclusions

This year is the 45th anniversary of the discovery of one of the most relevant proteins: p53. This phosphoprotein still has much to be studied, especially in elucidating the finer mechanistic details of its involvement in LUSC. It also remains a scientific challenge today to find effective drugs and new therapies in this field that are specifically aimed at counteracting the harmful effects of p53 in lung cancer cells. In the current personalized medicine era, fully comprehending the function of p53 and its variants in the behavior of lung cancer is crucial for shaping the diagnosis and treatment landscape of the disease.

## Acronyms

AKT	Protein kinase B	LUSC	Lung squamous cell carcinoma
ALK	Anaplastic lymphoma kinase	MAPK	Mitogen-activated protein kinase
AMPK	AMP-activated protein kinase	MDM2	Mouse double minute 2 homolog
AT1	Alveolar type 1	MDMX	Mouse double minute X
AT2	Alveolar type 2	mRNA	Messenger RNA
ATM	Ataxia telangiectasia mutated	mTOR	Mammalian target of rapamycin
ATR	Ataxia telangiectasia and Rad3-related protein	NGS	Next-generation sequencing
CDKN2A	Cyclin-dependent kinase inhibitor 2A	NSCLC	Non-small-cell lung cancer
CTD	C-terminal domain	OD	Oligomerization domain
ctDNA	Circulating tumor DNA	p53RE	p53-response elements
DBD	DNA-binding domain	PARP	Poly-ADP ribose polymerase
DLL3	Delta-like ligand 3	PD-1	Programmed cell death protein 1
DNA	Deoxyribonucleic acid	PD-L1	Programmed cell death 1 ligand 1
DNMT	DNA methyltransferase	PI3K	Phosphatidylinositol 3-kinase
EGFR	Epidermal growth factor receptor	PRD	Proline-rich domain
FLp53	Full-length p53	PTEN	Phosphatase and tensin homolog
GOF	Gain of function	Rap1A	Ras-associated protein 1A
HDAC	Histone deacetylase	Rb1	Retinoblastoma 1 protein
ICIs	Immune checkpoint inhibitors	RhoC	Ras homolog family member C
JNK	c-Jun N-terminal kinase	SCLC	Small-cell lung cancer
LC	Lung cancer	TAD	Transactivation domain
LOF	Loss of function	YAP	Yes-associated protein
LUAD	Lung adenocarcinoma		
